# Dynamic Characterization of a Low-Cost Fully and Continuously 3D Printed Capacitive Pressure-Sensing System for Plantar Pressure Measurements

**DOI:** 10.3390/s23198209

**Published:** 2023-09-30

**Authors:** Andrew T. Gothard, Jacob W. Hott, Steven R. Anton

**Affiliations:** Dynamic and Smart Systems Laboratory, Department of Mechanical Engineering, Tennessee Technological University, Cookeville, TN 38505, USA; atgothard42@tntech.edu (A.T.G.); jwhott42@tntech.edu (J.W.H.)

**Keywords:** orthopedics, 3D printing, flexible capacitive pressure sensor, plantar pressure measurement, dynamic sensor characterization

## Abstract

In orthopedics, the evaluation of footbed pressure distribution maps is a valuable gait analysis technique that aids physicians in diagnosing musculoskeletal and gait disorders. Recently, the use of pressure-sensing insoles to collect pressure distributions has become more popular due to the passive collection of natural gait data during daily activities and the reduction in physical strain experienced by patients. However, current pressure-sensing insoles face the limitations of low customizability and high cost. Previous works have shown the ability to construct customizable pressure-sensing insoles with capacitive sensors using fused-deposition modeling (FDM) 3D printing. This work explores the feasibility of low-cost fully and continuously 3D printed pressure sensors for pressure-sensing insoles using three sensor designs, which use flexible thermoplastic polyurethane (TPU) as the dielectric layer and either conductive TPU or conductive polylactic acid (PLA) for the conductive plates. The sensors are paired with a commercial capacitance-to-voltage converter board to form the sensing system. Dynamic sensor performance is evaluated via sinusoidal compressive tests at frequencies of 1, 3, 5, and 7 Hz, with pressure levels varying from 14.33 to 23.88, 33.43, 52.54, and 71.65 N/cm^2^ at each frequency. Five sensors of each type are tested. Results show that all sensors display significant hysteresis and nonlinearity. The PLA-TPU sensor with 10% infill is the best-performing sensor with the highest average sensitivity and lowest average hysteresis and linearity errors. The range of average sensitivities, hysteresis, and linearity errors across the entire span of tested pressures and frequencies for the PLA-TPU sensor with 10% infill is 11.61–20.11·10^−4^ V/(N/cm^2^), 11.9–31.8%, and 9.0–22.3%, respectively. The significant hysteresis and linearity error are due to the viscoelastic properties of TPU, and some additional nonlinear effects may be due to buckling of the infill walls of the dielectric.

## 1. Introduction

Gait analysis is a well-established field of study that explores human locomotion as it relates to patient health, which is helpful for rehabilitation after surgery and the diagnosis of various orthopedic conditions. The desire to reduce recovery time and improve the diagnosis of musculoskeletal disorders has driven the study of human locomotion and the development of measurement instruments such as multidirectional piezoelectric force plates, which give information about vertical and shear components of ground reaction forces of a patient during gait testing [1]. Pressure measurement floor systems have improved upon force plates by allowing for the collection of pressure distribution data for a patient’s foot during a gait test [2]. Plantar pressure distributions can provide important information on foot and ankle function and assist in determining musculoskeletal and neurological disorders [3,4,5]. A simple example of pressure distribution information assisting in diagnosis is demonstrated by Hsu et al., who used information on plantar pressure to determine whether a patient requires treatment for flat feet, which, left untreated, can lead to a greater risk of developing more severe conditions such as osteoarthritis [6,7]. Additionally, gait analysis data show the potential to diagnose other non-orthopedic diseases, such as Parkinson’s or Alzheimer’s [8]. However, floor-based pressure-sensing systems are limited because patients must travel to a facility capable of performing gait analysis. Typical gait tests are also time-consuming and occasionally strenuous for elderly patients or patients suffering from an orthopedic disease. Therefore, there has been a shift toward wearable pressure-sensing insoles rather than floor-based pressure-sensing systems for diagnosing movement disorders and assessing outcomes of surgical procedures [9]. Portable pressure-sensing insoles are commercially available and can be used at home by a patient to record and transmit gait data to a physician as daily activities are performed [10]. Using portable pressure-sensing insoles not only allows for footbed pressure distribution data to be captured but also prevents patients from having to undergo lengthy and expensive gait examinations, which results in reduced strain on patients. Additionally, portable pressure-sensing insoles allow more natural gait data to be collected for longer time periods than possible in a gait laboratory [11,12]. Previous groups have developed novel, portable footbed pressure-sensing systems. One particular sensor system of interest was created by Xu et al., who combined a pressure-sensing insole, a three-axis accelerometer, and a three-axis gyroscope and compass to record patient plantar pressure maps and movement information, which is sent to a smart device [13]. All data recorded on the smart device can then be uploaded to a cloud-based storage system for statistical analysis. The portable system by Xu et al. demonstrates the possibility of a multipurpose portable system that may be used to monitor a patient for both diagnosis and preventative purposes.

Many portable pressure-sensing insoles use specialized capacitive sensor arrays to determine plantar pressure due to the simple design and implementation of capacitive sensors [14,15,16,17]. However, existing insoles lack customizability or require high costs and/or fabrication times for the limited customizability offered. For instance, the system developed by De Guzman et al. is designed to be used as a low-cost system to monitor plantar pressure and collect data from children, but to change the size or shape of the insole, it is necessary to build and calibrate a new insole, requiring extra time and resources [18]. Similarly, Ho et al. developed a pressure-sensing insole system using capacitive sensors and conductive textiles that requires new models to be fabricated and calibrated for different sizes [19]. Further work has been done to improve the customizability of insoles by developing pressure-sensing insoles that can be adjusted to fit a patient’s shoe size through a trimming process [20]. Varoto et al. showed how a trimmable pressure-sensing insole could be integrated into a wireless system to monitor plantar pressure [21]. However, to keep the circuit intact, these insoles can only be adjusted to specific shoe sizes, thus limiting their use. Additionally, the shape of these insoles cannot be altered, which prevents patients with foot deformities, such as club feet, from using them.

Recently, 3D printing and the development of conductive and flexible filaments have presented the possibility of fabricating patient-specific pressure-sensing insoles with fully 3D printed capacitive sensors. Capacitive sensors are designed to compress when pressure is applied, which results in a decrease in the distance between the conductive plates. When the distance between the conductive plates decreases, the capacitance of the sensor increases, which can be measured and correlated to the pressure change via calibration. Other types of 3D printed pressure sensors exist, such as piezoelectric and piezoresistive sensors. This work chooses to focus on 3D printed capacitive sensors because they benefit from the broad availability of dual-extrusion commercial 3D printers and filaments that can be used to print capacitive sensors, which gives the potential for 3D printed capacitive sensors to be easily integrated into clinical settings. Previous work by Leigh et al. developed a novel conductive filament called “carbomorph” from which preliminary capacitive pressure sensors were printed to sense when a material flexed [22]. This study showed the possibility of using a conductive filament to sense pressure changes by measuring capacitance changes. Other work has also demonstrated various hybrid 3D printing methods (i.e., 3D printing in combination with other non-3D printing techniques) to create capacitive pressure-sensing systems [23,24,25,26,27,28]. Ntagios et al. used multiple filament types and printable inks to create tactile pressure-sensing systems for a robotic hand [25]. Their work demonstrated the customizability of the shape and material of 3D printed pressure sensors. Jia et al. created a method to fabricate capacitive pressure sensors that combined fused-deposition modeling (FDM) 3D printing and digital light processing (DLP) 3D printing [23]. Saari et al. developed pressure sensors by encapsulating wire in a tight spiral pattern within 3D printed thermoplastic elastomers [27]. Another work by Valentine et al. developed a hybrid method that printed a thermoplastic polyurethane (TPU) matrix on an insole material and used a pick-and-place machine to place commercial capacitive sensors in the TPU matrix [28]. Their work demonstrated a flexible pressure-sensing insole that could be printed to patient-specific sizes and used in biomedical applications. This work shows the potential for utilizing 3D printing to customize the size and pattern of the pressure sensor array for footbed pressure-sensing insoles. However, the method is limited by a pick-and-place machine, which would increase cost and decrease accessibility. Furthermore, the shape of the insole body was not customized to each patient’s foot. Ntagios et al. demonstrated a method to create a hybrid 3D printed capacitive pressure sensor through a print-pause-print method, which involved printing a base electrode, pouring a dielectric, and printing the top electrode [26].

A fully 3D printed, capacitive-sensing insole would provide a cost-effective means to create insoles that could be customized to fit the shape and size of a patient’s foot while also controlling the sensor placement within the insole. Previous work by Voronov and Dovgolevskiy demonstrated a method to design a customized insole based on a 3D scan of a patient’s foot and an adversarial neural network [29]. A similar approach could be used to customize the shape of a pressure-sensing insole to match a patient’s footbed by scanning the patient’s plantar surface into a point cloud, converting it into an STL file, inserting a 3D printed sensor, and developing wire geometry in the insole. The geometry could then be converted into a G-code and printed in a dual-extrusion printer. Since each sensor would be printed simultaneously with the insole, other hybrid methods would not be needed to build the sensor network. Currently, there are no fully developed methods for creating, testing, and validating fully 3D printed sensors that are seamlessly integrated into a printed insole. Past work performed by Schouten et al. developed a novel 3D printed capacitive pressure sensor design using flexible filament for both the dielectric and conductive plate materials [30]. While these sensors showed an acceptable change in capacitance for pressure-sensing applications, the sensors also showed considerable drift, likely caused by creep. Additionally, the sensors were not characterized dynamically. Schouten et al. later developed a fully 3D printed sensor to measure both shear and normal forces, but the sensor requires additional assembly after printing [31]. A recent study by Samarentsis et al. designed a highly customizable, fully 3D printed insole that can be customized to a specific patient and printed quickly and cost-effectively [32]. Samarentsis et al.’s design comprises three parts that are printed separately. The parts are assembled and connected to an Arduino microcontroller to monitor plantar pressure. While this is a significant step forward for customizable 3D printed pressure-sensing insoles, the insole design requires manual assembly. Additionally, the sensors were only tested at frequencies up to approximately 1.16 Hz. Another work by Ntagios and Dahiya created a fully 3D printed insole, but this design also required several assembly steps to place wire within the system [33].

The current work aims to address some of these limitations through the development and dynamic characterization of low-cost fully and continuously 3D printed capacitive sensors at frequencies and load levels typical for adult footbed pressure-sensing applications (approximately 0 to 10 Hz and 0 to 35 N/cm^2^ respectively) [34,35,36]. The fully and continuously 3D printed nature of the sensors allows them to be seamlessly integrated into pressure-sensing insoles in the future. Three sensor designs are developed and evaluated in this work: flexible TPU as the dielectric layer and either conductive flexible TPU or conductive polylactic acid (PLA) for the conductive plates. The sensors are printed using a dual-filament FDM 3D printer. Dynamic characterization of sensor performance is then carried out via a series of experimental tests. Five sensors of each type are tested. A commercial capacitance-to-voltage converter circuit is employed to convert sensor capacitance to measurable voltage, and an electrodynamic uniaxial load frame is used to excite the sensor at 1, 3, 5, and 7 Hz, with pressure levels varying from 14.33 to 23.88, 33.43, 52.54, and 71.65 N/cm^2^ at each frequency. This excitation range encompasses most of the typical frequency band observed in daily activity and up to twice the normal maximum pressure to ensure survivability. Sensor calibration curves are generated for every sensor at each combination of input frequency and pressure and are used to compute and report average sensitivity, average linearity error, and average hysteresis error. The main contributions of this work in the area of 3D printed capacitive sensors include: (1) the sensors are fully and continuously 3D printed, thus do not require assembly, which is less commonly explored in the literature compared to hybrid methods; and (2) the sensors are dynamically characterized at higher frequencies than many of the previous works. Additionally, this work also explores the performance of conductive TPU vs. PLA filaments as sensor electrodes.

## 2. Sensor Development

Sensors in the current work are designed as parallel-plate capacitive pressure sensors, which consist of two conductive parallel plates (electrodes) with a dielectric material separating them. When pressure is applied to the sensor, it compresses, and the capacitance of the sensor varies according to the following expression:

(1) $$\Delta C=\frac{\epsilon A}{\Delta x}\;,$$

where ∆C is the change in capacitance, ϵ is the permittivity of the dielectric, A is the overlapping area of the two conductive plates, and Δx is the change in distance between the conductive plates. For the capacitive pressure sensor design to be effective, the sensor must have a sufficient change in Δx to develop a measurable capacitance change. Additionally, the capacitive pressure sensors must be sufficiently slender to fit within a developed insole while being adequately pliable to maintain comfort and decrease any adverse effects on the user’s gait cycle. For these reasons, three different 3D printed sensor designs are developed with a flexible TPU dielectric and either a conductive PLA or TPU for the electrodes. These three designs are dynamically tested to study the effects of changing the material used for the electrodes and varying the infill density of the dielectric on both the print quality and dynamic performance of the sensor. The first two types of sensors are created with Protopasta conductive PLA filament (ProtoPlant Inc., Vancouver, WA, USA) for the electrodes and NinjaTek 85A nonconductive TPU filament (Fenner Precision Polymers, Lititz, PA, USA) for the dielectric. The first developed sensor type has a 10% infill density for the TPU dielectric, referred to as a PLA-TPU-10 sensor. The second sensor type has a 30% infill density for the TPU dielectric, referred to as a PLA-TPU-30 sensor. The third sensor type uses NinjaTek Eel, a conductive TPU filament, for the electrodes and NinjaTek 85A TPU for the dielectric. The dielectric infill density for the third sensor type is 30%. The third sensor type is referred to as an Eel-TPU-30 sensor. The conductive plates for all three sensor types use an infill of 100%.

**Figure 1:**
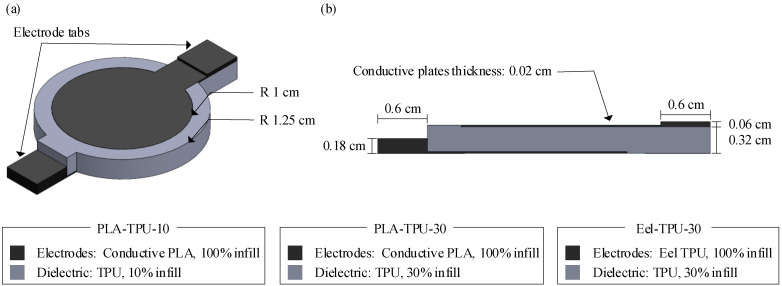
Fully and continuously 3D printed capacitive sensor design showing (**a**) an isometric view and (**b**) a cross-sectional view, and providing details of the materials used to fabricate each sensor type.

**Figure 2:**
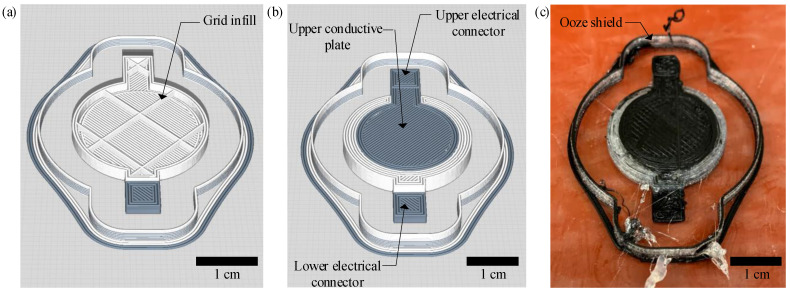
PLA-TPU-10 sensor design showing (**a**) cross-sectional view, (**b**) normal view, and (**c**) fabricated sensor.

The overall sensor design can be seen in Figure 1. The sensor geometry is developed using SOLIDWORKS (Dassault Systemes SolidWorks Corp., Waltham, MA, USA), a computer-aided design (CAD) software package, and has an overall radius of 1.25 cm and a conductive plate radius of 1 cm, which gives an active sensor area of 3.14 cm^2^. The active sensor area is selected so that multiple sensors can be placed in an adult-sized insole to capture the footbed pressure of the anatomical regions of the footbed. The uncompressed distance between capacitive plates is 0.3 cm. The 0.3 cm thickness is chosen because it reasonably allows the sensor to be integrated into an insole while being thick enough to allow the dielectric infill density to be varied. Electrode tabs are included to facilitate the attachment of electrical test leads; however, these tabs would be replaced with 3D printed electrical traces in a fully 3D printed sensing insole. The sensor geometry is exported from SOLIDWORKS as an STL file and uploaded to CURA Lulzbot Edition (Fargo Additive Manufacturing Equipment 3D, LLC, Fargo, ND, USA), slicing software used to generate G-code. The resulting G-code file is then uploaded to a LulzBot TAZ 6 printer (Fargo Additive Manufacturing Equipment 3D, LLC, Fargo, ND, USA) equipped with a LulzBot Dual Extruder V2 tool head. The tool head is modified to print flexible filaments. Multiple trials were performed to find the optimal print parameters for each sensor type. The final print parameters can be seen in Table 1. Any settings not included in the table are left at the default setting in CURA. The previously mentioned infill settings for the dielectric are determined with preliminary prints where the infill is gradually decreased to increase the sensor’s sensitivity. The initial prints show that the dielectric infill percentage can be reduced to 10% for sensors constructed with Protopasta conductive PLA plates and still maintain a usable sensor print quality. However, for the sensors made with NinjaTek Eel plates, the lowest dielectric infill percentage achieved is 30%. PLA-TPU-30 sensors are created to compare with the PLA-TPU-10 and Eel-TPU-30 sensor types to observe the effect of both electrode material and infill percentage on performance. An example of the PLA-TPU-10 sensor design and printed sensor can be seen in Figure 2. It should be noted that printing temperature and printing speed for TPU filaments are set to remain constant throughout the print for each sensor type to increase the print quality of the sensors. Ooze shields are used to minimize filament mixing. 

The cost for each sensor is also estimated. At the time of conducting this research, the costs for a 1 kg role of Protopasta conductive PLA, NinjaTek 85A, and NinjaTek Eel filaments are 90, 88, and 150 U.S.dollars (USD), respectively. PLA-TPU sensors require 2 g of Protopasta conductive PLA and 2 g of NinjaTek 85A filament, which gives a total cost estimate of 0.36 USD/sensor. TPU-TPU sensors require 2 g of NinjaTek Eel and 2 g of NinjaTek 85A, which gives a total cost estimate of 0.48 USD/sensor. It should be noted that the CURA software provides filament mass estimates that are used for these calculations. These cost estimates demonstrate the low cost of the sensors in this work.

## 3. Experimental Methods

### 3.1. Test Setup—Dynamic Characterization of the 3D Printed Capacitive Pressure-Sensing System 

**Figure 3:**
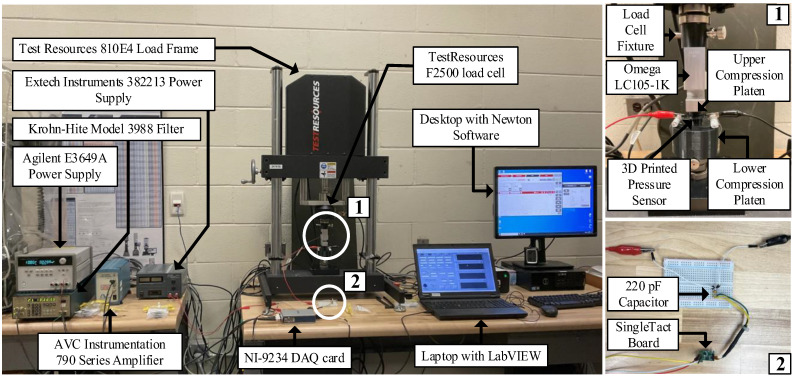
Experimental test setup for dynamic characterization of the 3D printed capacitive pressure-sensing system.

To quantify the dynamic response of the three sensor types, a controlled cyclic pressure must be applied to the sensor, and the capacitance change in the sensor (on the order of picofarads) must be converted to a measurable voltage and sampled. The capacitance change for each sensor is converted to a voltage signal using a commercial SingleTact capacitance-to-voltage converter board (PPS UK Limited, Glasgow, UK), which is powered by 5 V from an Extech Instruments 382213 DC power supply (Extech Instruments, Nashua, NH, USA). A 220 pF capacitor is wired in parallel with the 3D printed capacitive sensor in order to bring the baseline capacitance up to a similar value as commercial SingleTact capacitive sensors, for which the converter board is designed. A TestResources 810E4 electrodynamic load frame (TestResources, Inc., Shakopee, MN, USA) is used to apply sinusoidal loads at various frequencies to each sensor. Custom compression platens and a fixture are designed and 3D printed for the load frame. A lower compression platen is affixed to the frame to hold the sensor in place during the test and facilitate secure attachment of the electrodes. An upper compression platen is fastened to an Omega LC105-1k load cell (Omega Engineering Inc., Norwalk, CT, USA) in order to transfer the compressive load only to the active area of the sensor. The force input is measured with the Omega load cell that is mounted between the upper compression platen and load frame. The connection to the load frame is made using a custom 3D printed fixture. The Omega load cell is oriented in series with a TestResources F2500 load cell with the aforementioned fixture and is powered with 10 V from an Agilent E3649A DC power supply (Agilent Technologies, Inc., Santa Clara, CA, USA). The TestResources load cell is used by the load frame control software (Newton) to provide closed-loop force control, whereas the auxiliary Omega load cell is used to provide a force signal that can be time-synchronized with the sensor output signal (note: the TestResources load cell output is not accessible outside of the Newton controller). A National Instruments NI-9234 DAQ card placed in a cDAQ-9171 single slot chassis (National Instruments Corp., Austin, TX, USA) synchronously records the input force from the Omega load cell and the output voltage from the SingleTact capacitance-to-voltage converter board at a sampling rate of 1652 Hz. The original signal output from the Omega load cell for the loads investigated in this work is too small (−0.00674 mV/N, in compression) to be accurately measured by the NI DAQ; therefore, the signal is scaled by a factor of −50 with an AVC Instrumentation 790 series inverting amplifier (PCB Piezotronics Inc., Depew, NY, USA) and filtered with a Krohn-Hite Model 3988 filter (Krohn-Hite Corp., Brockton, MA, USA). The Krohn-Hite filter is configured as a low-pass Butterworth filter with a cutoff frequency of 30 Hz to eliminate any amplified noise in the signal. The test setup can be seen in Figure 3.

### 3.2. Phase-Lag Characterization of the Measurement System 

The various signal conditioning components in the measurement system (i.e., amplifier, filter, capacitance-to-voltage converter) impart a phase delay in the signals as measured by the data acquisition system. Therefore, before dynamic sensor testing can be carried out, it is necessary to quantify the phase lag of both the force measurement system and the capacitance measurement system so that proper alignment of the input pressure and output voltage signals can be performed in the time domain.

#### 3.2.1. Force Measurement Phase Lag

**Figure 4:**
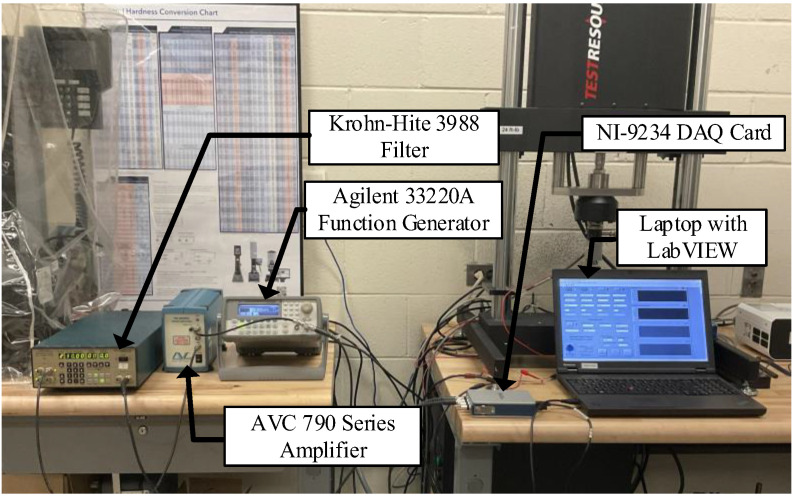
Experimental test setup for force measurement phase-lag quantification.

The force measurement system consists of the Omega load cell, the AVC Instrumentation inverting amplifier, and the Krohn-Hite filter. The phase lag of the force measurement system is determined by first generating a voltage that mimics the output voltage signal of the Omega load cell during a sinusoidal compression test with an Agilent 33220A function generator. Note that the simulated signal is slightly larger in terms of amplitude (~20 mV) compared to the load cell’s unaltered output signal due to the amplitude limitations of the function generator used; however, the phase lag of the force measurement system is not expected to change due to the difference in amplitude. The simulated signal is split into two separate signals: the first signal is unaltered, and the second is routed through the AVC Instrumentation amplifier and the Krohn-Hite filter. Both signals are then captured synchronously by the NI-9234 DAQ card at a sampling rate of 1652 Hz. All settings for the filter and amplifier are specified in Section 3.1. The resulting test setup can be seen in Figure 4. 

**Figure 5:**
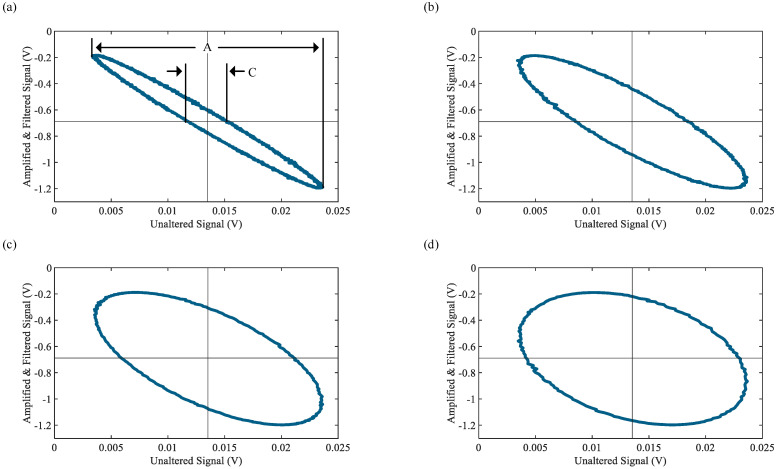
Resulting Lissajous patterns for (**a**) 1, (**b**) 3, (**c**) 5, and (**d**) 7 Hz used to quantify phase lag in the force measurement system.

**Figure 6:**
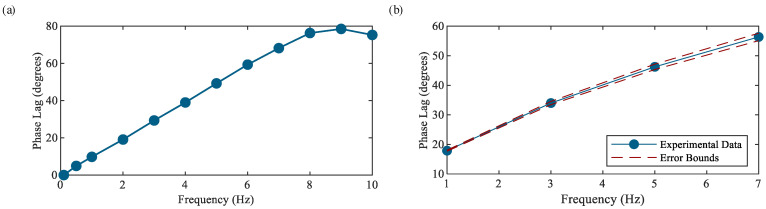
Phase lag in (**a**) force measurement and (**b**) capacitance measurement systems as a function of frequency.

To encompass the frequencies used in the dynamic sensor characterization, several cycles of the signals are measured within the frequency range of 0.1 to 10 Hz. After testing, the frequency of the simulated load cell signal is verified at 1, 3, 5, and 7 Hz using a fast Fourier transform (FFT). Results of the FFT show a maximum error of 0.57% for the targeted frequencies. The phase lag between the two signals is determined using Lissajous patterns. Lissajous patterns neglect the time domain of both signals and plot the amplitude of each signal at a given time point on a 2D plot, thus yielding a graphical means for calculating the phase difference between two simultaneously sampled signals. Phase lag for a single random cycle of both signals is calculated with the following expression [37]:

(2) $$\left|\theta_{1}-\theta_{2}\right|=\left|{sin}^{-1}\left(\frac{C}{A}\right)\right|,$$

where C is the distance between the signal’s intersections at the signal’s *y*-axis average, A is the distance between the maximum and minimum values occurring along the *x*-axis, and $\left|\theta_{1}-\theta_{2}\right|$ is the phase lag between the signals in radians. The Lissajous patterns captured for the simulated signal at frequencies of 1, 3, 5, and 7 Hz can be seen in Figure 5. The quantities C and A are also shown graphically in Figure 5a. The resulting phase shift plot of the force measurement system at all tested frequencies can be seen in Figure 6a. It shows that significant phase lags, up to a maximum of approximately 80 degrees, are observed; thus, it is important to correct for the phase lag caused by the force measurement system during data processing when performing dynamic sensor characterization.

#### 3.2.2. Capacitance Measurement Phase Lag

To determine the phase lag occurring in the capacitance measurement system (i.e., the SingleTact capacitance-to-voltage converter board), the TestResources load frame is used to apply a sinusoidal compressive load at frequencies of 1, 3, 5, and 7 Hz at a pressure level of 75 N/cm^2^ to a commercial SingleTact capacitive sensor. The sensor has an active area of 2.512 cm^2^; thus, a force of 188 N is applied to achieve the desired pressure. The resulting change in capacitance is then converted into a voltage signal by the SingleTact capacitance-to-voltage converter board and is captured by the NI-9234 DAQ card at a sampling rate of 1652 Samples/s; note that an increase in capacitance (i.e., a capacitive sensor under compression) results in a positive output voltage. It should be noted that the SingleTact capacitance-to-voltage converter requires self-calibration each time a new sensor is connected, which is achieved by ensuring the new sensor is under zero load and cycling power to the board, and the system uses approximately 0.5 V as its nominal output voltage. The amplified and filtered voltage signal from the Omega load cell is captured synchronously by the same DAQ device and used to verify the frequency of the load frame via an FFT and determine phase lag. The maximum frequency error for tested frequencies is found to be 0.53%. Once all frequencies have been verified, the Omega load cell voltage signal is time-shifted according to the previously determined phase lag given in Figure 6a. The time shift for a particular input frequency ω is found using the following expression:

(3) $$t_{d}(\omega )=\frac{\left|\theta_{1}-\theta_{2}\right|\left(\omega \right)\cdot T(\omega )}{360{}^{\circ}},$$

where $t_{d}$ is the time shift, $\left|\theta_{1}-\theta_{2}\right|$ is the phase lag in degrees, and T is the period of the signal. Lissajous patterns are once again utilized to find the phase lag in the capacitance measurement system per Equation (2). The resulting phase shift plot can be seen in Figure 6b. It can be observed that significant phase lags, up to a maximum of approximately 55 degrees, are observed; thus, it is important to correct for the phase lag caused by the capacitance-to-voltage converter board when performing dynamic sensor characterization. It is worth noting that the uncertainty in the calculated phase lag for the capacitance measurement system depends on the response time of the commercial SingleTact capacitive sensor. The SingleTact capacitive sensor has a response time of less than or equal to 1 ms, so the uncertainty in the performed calculations is expected to be ±0.5 ms. This error bound has been converted to degrees using Equation (3) and is shown in Figure 6b.

### 3.3. Sensitivity Characterization of the Omega Load Cell

**Figure 7:**
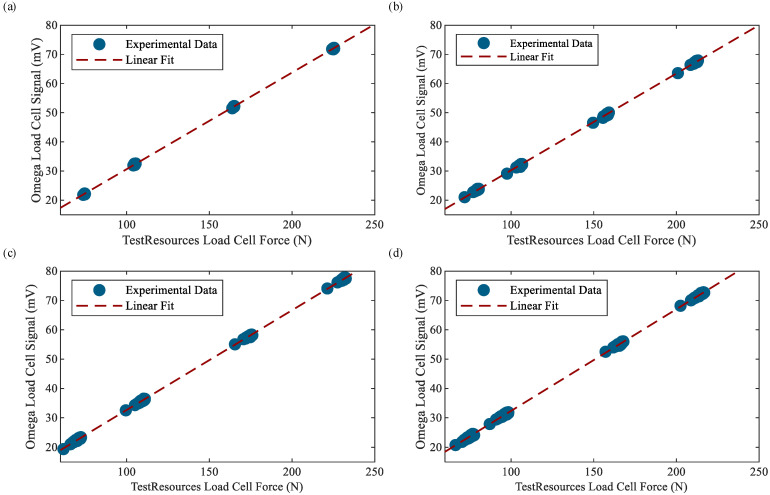
Calibration plots for the amplified Omega load cell at (**a**) 1, (**b**) 3, (**c**) 5, and (**d**) 7 Hz.

As mentioned before, the Omega load cell has a sensitivity of −0.00674 mV/N (when in compression), which is amplified by a factor of −50; thus, the resulting sensitivity is approximately 0.337 mV/N. This provides an appropriate voltage level for data acquisition at the prescribed loads. The manufacturer, however, does not provide an operational frequency range. Therefore, the sensitivity of the Omega load cell at frequencies of 1, 3, 5, and 7 Hz must be determined to account for the effects of the amplifier, filter, and any frequency-dependent load cell effects. Characterizing the load cell sensitivity at different frequencies is performed by utilizing the data previously collected during phase-lag testing of the capacitance measurement system. Force data from the TestResources load cell (measured by the load frame controller and considered the ground truth) is first inverted to match the amplified Omega load cell sign convention (compression is positive). Peaks from the resulting force signal and the amplified and filtered Omega load cell voltage signal are found and plotted against each other on a 2D plot with force on the *x*-axis and Omega load cell voltage on the *y*-axis. Linear regression is performed in MATLAB (The MathWorks, Inc., Natick, MA, USA) to compute the average sensitivity for each frequency tested. Sensitivities can be seen in Table 2, and the resulting plots can be seen in Figure 7. It can be observed that the Omega load cell sensitivity varies slightly with frequency; thus, the frequency-dependent sensitivities are used to scale collected voltage data from the Omega load cell to units of newtons depending on the excitation frequency of the test.

### 3.4. Test Procedure—Dynamic Characterization of the 3D Printed Capacitive Pressure-Sensing System 

With the phase lag of both measurement systems and the frequency-dependent sensitivity of the load cell determined, the three sensor types are dynamically tested. Five sensors of each type are tested. Each sensor is evaluated via sinusoidal compressive pressure tests of varying frequency and peak pressure values. For each test, the pressure is ramped to a preload of 14.33 N/cm^2^ and cycles between the preload and the given peak pressure level. The peak pressure levels tested include: 23.88, 33.43, 52.54, and 71.65 N/cm^2^, and frequencies tested include: 1, 3, 5, and 7 Hz. The pressures and frequencies are chosen to span the typical pressure and frequency range for walking, which are approximately 0 to 10 Hz and 0 to 35 N/cm^2^, respectively [34,35,36]. Additionally, the pressure levels are tested to nearly double the typical maximum pressure value for walking to characterize the sensors for ranges reasonable for higher impact activities such as running. The maximum frequency is 7 Hz due to limitations of the PID force control of the TestResources load frame used for testing. It should be noted that the minimum measurable pressure value for commercial footbed pressure sensors is near 0 N/cm^2^. For instance, the commercial Pedar footbed pressure-sensing insole has a pressure range of 1.5 to 60 N/cm^2^ [38]. For the sensors developed in this work, 14.33 N/cm^2^ is used as the preload value based on preliminary testing results, in which 14.33 N/cm^2^ is found to be the lowest pressure level reasonably measured by the pressure-sensing system. Additionally, the lowest pressure range tested is between 14.33 and 23.88 N/cm^2^ because the sensing system is not sensitive enough to detect any smaller changes in pressure. In total, there are 16 tests performed for each sensor. Data collected from the capacitance measurement system are synchronized with data collected from the force measurement system using the phase lags found in Section 3.2.1 and Section 3.2.2. This is accomplished by subtracting the time shift from the array of time data and deleting negative time values. Next, the Omega load cell voltage is converted to newtons according to the frequency-dependent sensitivities reported in Table 2, and pressure is calculated by dividing the applied force by the area of the compression platen (3.14 cm^2^).

## 4. Results and Discussion

After data collection and postprocessing, the performance of the sensors is analyzed and then discussed. It should be noted that only four of the five sensors of the PLA-TPU-10 sensor type are used in the sensor analysis due to a print defect found in one of the sensors during testing.

### 4.1. Dynamic Sensor Characterization Results

**Figure 8:**
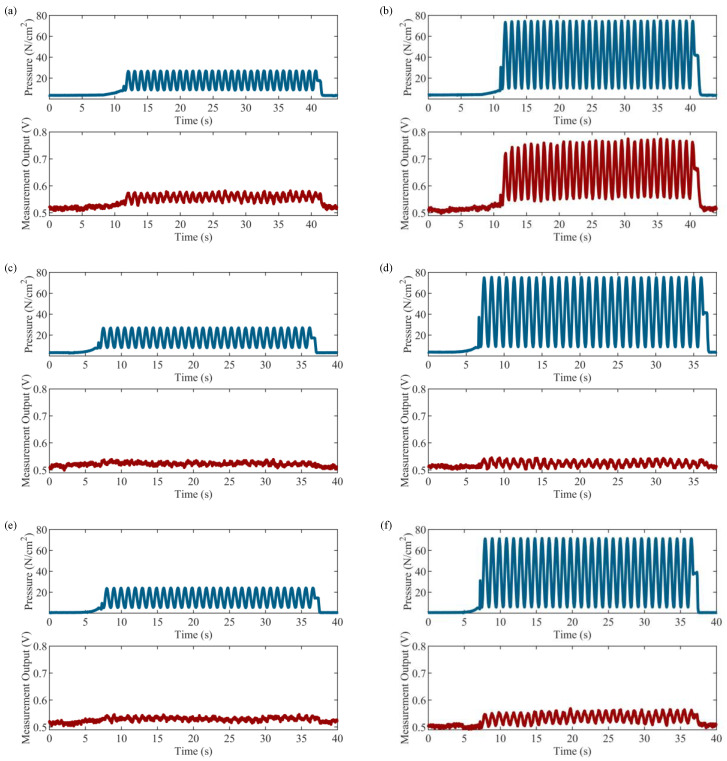
Representative pressure input and sensor output time histories at 1 Hz for the PLA-TPU-10 sensor with input pressure of (**a**) 23.88 and (**b**) 71.65 N/cm^2^; for the PLA-TPU-30 sensor with input pressure of (**c**) 23.88 and (**d**) 71.65 N/cm^2^; and for the Eel-TPU-30 sensor with input pressure of (**e**) 23.88 and (**f**) 71.65 N/cm^2^.

First, the pressure input and measurement output time histories are plotted and used to quantify the average output voltage range and signal-to-noise ratio (SNR) of the sensors when paired with the SingleTact capacitance-to-voltage converter board. Representative graphs showing the pressure input and the resulting sensor output time histories for 23.88 and 71.65 N/cm^2^ tests at 1 Hz for each of the three sensor types can be seen in Figure 8. The average output voltage range is computed as the difference between the maximum output voltage value and the minimum output voltage value (which, as previously mentioned, is around 0.5 V as set by the capacitance-to-voltage converter) at a given pressure and frequency, averaged across all five sensors for each sensor type. The average SNR is computed by calculating the SNR in decibels (dBs) at a given pressure and frequency, given by $SNR(dB)=20\log\left(V_{s,RMS}/V_{n,RMS}\right)$, where $V_{s,RMS}$ is root-mean square (RMS) of the sensor voltage output across all complete cycles in a given test, and $V_{n,RMS}$ is the RMS of the first 1.5 s of static noise that occurs during the test, averaged across all five sensors for each sensor type. The resulting average output voltage range and SNR values can be seen in Table 3. From the results, it can be observed that the PLA-TPU-10 sensor provides 1.5–3 times greater output voltage range than the PLA-TPU-30 and Eel-TPU-30 sensors, which perform similarly to one another. The larger output voltage range of the PLA-TPU-10 sensor corresponds to an increase in SNR, which is also observed in the results. Between the PLA-TPU-30 and Eel-TPU-30 sensors, the Eel electrode layer appears to provide a slight increase in performance over the PLA electrode layer. Furthermore, it can be observed that for each sensor the SNR naturally increases with increasing input pressure; what is interesting to observe is that, generally, the SNR tends to decrease with an increase in input frequency, particularly at higher pressure levels. This may be due to frequency-dependent hysteresis effects in the sensors, which are explored next.

**Figure 9:**
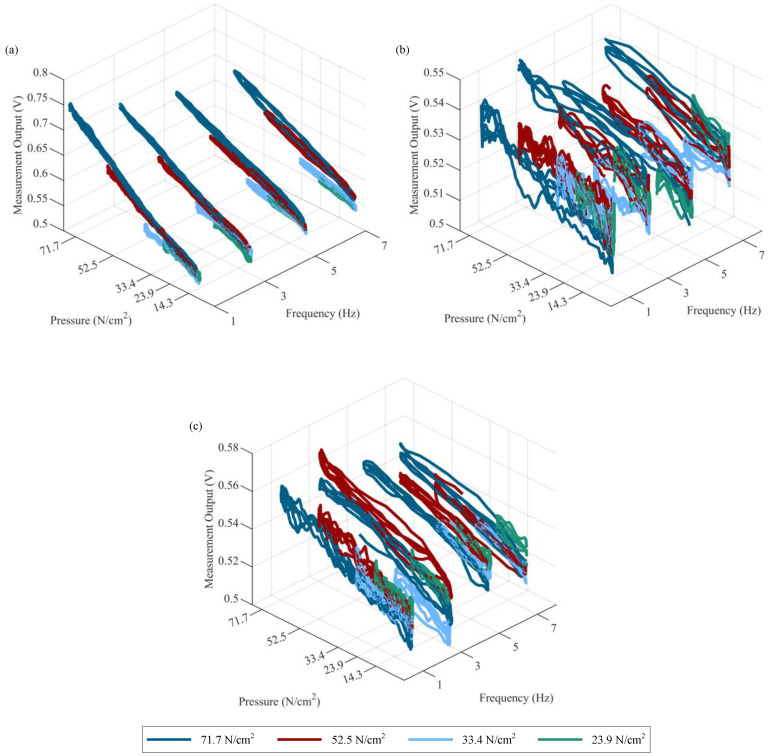
Representative sensing-system calibration curves (limited to three cycles for readability) for (**a**) PLA-TPU-10, (**b**) PLA-TPU-30, and (**c**) Eel-TPU-30 sensor types.

**Figure 10:**
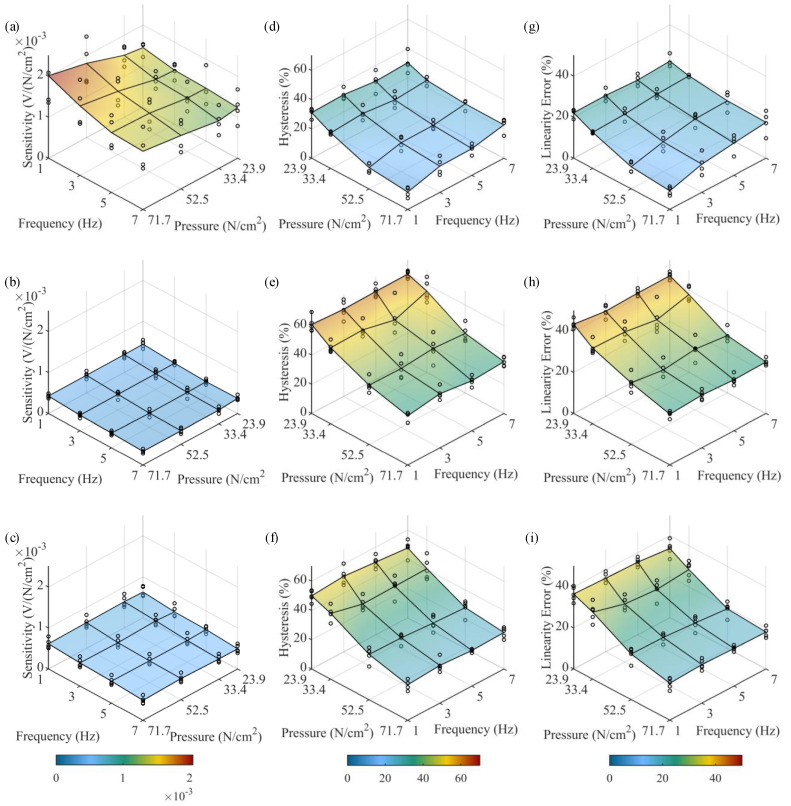
Sensor performance plots showing sensitivity for (**a**) PLA-TPU-10, (**b**) PLA-TPU-30, and (**c**) Eel-TPU-30 sensor types; hysteresis error for (**d**) PLA-TPU-10, (**e**) PLA-TPU-30, and (**f**) Eel-TPU-30 sensor types; and linearity error for (**g**) PLA-TPU-10, (**h**) PLA-TPU-30, and (**i**) Eel-TPU-30 sensor types. Black circles represent individual sensors, and colored surface plots represent averages across all sensors.

Next, further analysis is performed by computing sensitivity, hysteresis, and linearity error values for each sensor at each pressure and frequency level. This is accomplished by first extracting 20 single-cycle calibration curves from the first 20 full-loading cycles (Figure 8) for each sensor at each pressure and frequency level. Representative calibration curves are shown in Figure 9. Second, sensitivity, hysteresis, and linearity error values are computed for each of the 20 cycles and then averaged; these values are plotted as black circles in Figure 10 to help understand any sensor-to-sensor variability present. Lastly, average values for sensitivity, hysteresis, and linearity error are computed at each pressure and frequency level by averaging the results from each sensor type; these values are reported in Table 4 and shown as surface plots in Figure 10 to help understand any dependence on input pressure and frequency. From the results of sensitivity, the range of average sensitivities across the entire span of tested pressures and frequencies for the PLA-TPU-10, PLA-TPU-30, and Eel-TPU-30 sensors is $11.61–20.11\cdot 10^{-4},\;3.30–4.42\cdot 10^{-4},\;\mathrm{and}\;4.85–6.40\cdot 10^{-4}\;V/(N/cm^{2})$, respectively. It can be observed that the PLA-TPU-10 sensors have 2–4 times higher average sensitivity compared to the PLA-TPU-30 and Eel-TPU-30 sensor types, while the Eel-TPU-30 sensor has a slightly higher average sensitivity than its PLA-TPU-30 counterpart. Moreover, the average sensitivity varies slightly for different pressure and frequency levels, particularly for the PLA-TPU-10 sensors, which show an easily observed increase in average sensitivity as pressure increases and as frequency decreases. Overall, there is significant variability in sensitivity between sensors printed to the same specifications, particularly for the PLA-TPU-10 sensors. From the results of hysteresis, significant hysteresis is present for all three sensor types, with averages ranging from 11.9–31.8, 33.4–60.9, and 24.3–51.7% for the PLA-TPU-10, PLA-TPU-30, and Eel-TPU-30 sensors, respectively. The average hysteresis is significantly lower for the PLA-TPU-10 sensors compared to the PLA-TPU-30 and Eel-TPU-30 sensor types. Furthermore, the average hysteresis for all sensor types tends to decrease with an increase in pressure and shows little dependence on frequency. Overall, there is some sensor-to-sensor variability in hysteresis for all sensor types. From the results of linearity error, significant linearity error is present for all three sensor types, with averages ranging 9.0–22.3, 24.6–43.5, and 18.0–36.5% for the PLA-TPU-10, PLA-TPU-30, and Eel-TPU-30 sensors, respectively. The trends are similar to those of hysteresis, with the PLA-TPU-10 sensors showing significantly lower average linearity error compared to the PLA-TPU-30 and Eel-TPU-30 sensor types, the average linearity error tending to decrease with an increase in pressure and having little dependence on frequency for all sensor types, and some sensor-to-sensor variability present for all sensor types.

### 4.2. Discussion

Reflecting on the results, several interesting points of discussion arise. First, it is easily observed that all sensors exhibit significant hysteresis and nonlinearity, together with some pressure- and frequency-dependent behavior. This is due to the viscoelastic properties of the soft polymeric materials used to fabricate the sensors. The dielectric portion of all the sensor types is TPU, and it is well-known that TPU is viscoelastic and demonstrates hysteresis and nonlinearity under compression [39]. Additionally, the authors hypothesize that the walls of the infill structure may buckle during compression, which may contribute to the linearity errors observed. Second, in regard to comparing the performance of the three sensor types, the PLA-TPU-10 sensor type has the largest sensitivity, voltage output range, and SNR, and the smallest hysteresis and linearity error, but the largest variability in sensitivity. The increase in sensitivity, voltage output range, and SNR are likely due to the decrease in infill percentage resulting in an increase in axial mechanical compliance, which would result in a larger capacitance change for a given input pressure, per Equation (1). The decreases in hysteresis and linearity error are likely due to the decrease in volume of viscoelastic TPU material used in the sensor. The increase in variability may also be due to the decrease in volume (infill percentage) of the TPU dielectric, which may result in less consistency in print quality. Comparing the PLA-TPU-30 sensor type to the Eel-TPU-30 sensor type, performance is generally similar. This is likely due to the fact that these sensor types both utilize the same dielectric material and infill percentage, and thus their dielectric layers have similar mechanical compliance. Based on all of the observed data, we hypothesize that the dielectric layer dominates sensor behavior. In terms of the conductive layer, the Eel electrode appears to provide a slight increase in sensitivity, voltage output range, and SNR over the PLA electrode; while the exact cause of this phenomenon is unknown, it may be attributed to differences in electrode layer properties, including conductivity, axial compliance, Poisson’s ratio (which could cause differences in radial compliance), etc., and/or differences in print quality.

In order for the sensors developed in this work to be integrated into footbed pressure sensing or other orthopedic applications, it is important to first define the requirement of the measurement system in terms of input parameters (e.g., pressure and frequency range) and measurement resolution. The SNR of the system dictates a minimum resolvable dynamic input pressure. To thoroughly characterize overall sensor performance, several metrics are calculated for each sensor type at various minimum SNR levels. First, minimum resolvable input pressure and frequency ranges are determined and reported. Second, ranges for average sensitivity, hysteresis, and linearity error are calculated and reported across the corresponding resolvable pressure and frequency ranges to help understand any dependence on input pressure and frequency. Lastly, to help understand any sensor-to-sensor variability, percent deviations of sensitivity, hysteresis, and linearity error for each individual sensor are calculated with respect to the mean value at each combination of input pressure and frequency. Then, 90% confidence intervals are calculated and reported for the resulting percent deviation values. Results for all computed metrics are shown in Table 5. From the results, several observations can be made. First, the resolvable pressure and frequency ranges decrease as the requirements on minimum SNR increase. Next, the PLA-TPU-10 performs best in terms of resolvable range due to its higher sensitivity when compared to the other sensor types. Finally, the maximum hysteresis and linearity error decrease as the minimum SNR increases. To understand why, consider the fact that at low pressure levels there is an increase in measurement uncertainty due to noise; inclusion of such values in the hysteresis and linearity error calculation cause the resulting maximum hysteresis and linearity error to increase. Overall, the results presented in Table 5 can help determine appropriate future use of the sensors based on the minimum SNR required by the particular application.

Finally, it is worthwhile to consider the performance of the sensors developed in the current work to other 3D printed capacitive pressure/force sensors related to orthopedics; a comparison can be seen in Table 6, in which sensor performance, evaluation range, and materials and fabrication methods are presented. A direct comparison of sensitivity values is difficult because sensitivity values in previous works measure a change in capacitance directly and normalize it by a base capacitance, giving the units of $(\Delta F/F_{0})/kPa$, which simplifies to ${kPa}^{-1}$. The current work uses a capacitance-to-voltage converting circuit, which gives a voltage output and therefore sensitivities are given as $V/(N/{cm}^{2})$. In regard to quantifying hysteresis, only two of the previous studies reported hysteresis, with values around 10%. As for linearity error, no other works evaluated this characteristic. Additionally, the sensors in the current work are characterized over the broadest frequency range, with other works either only performing quasistatic testing or, at most, testing over a frequency range of 0.5–1.167 Hz. In terms of tested pressure range, most other works only explore significantly lower pressure (0–5 $N/{cm}^{2}$), with one other work having a broader pressure range of 4.15–87.24 $N/{cm}^{2}$. Finally, the sensors in this work are both continuously 3D printed and fully 3D printed (i.e., do not use hybrid 3D printing methods), which is not explored in any of the other orthopedic-related research works.

## 5. Conclusions

This study investigates the development and dynamic characterization of fully and continuously 3D printed capacitive sensors for use in customizable footbed pressure-sensing insoles. Three flexible 3D printed capacitive pressure sensor designs are presented and tested in this work, which include a polylactic acid (PLA)-conductive-plate/thermoplastic polyurethane (TPU)-dielectric sensor design with 10% dielectric infill (PLA-TPU-10), a PLA-conductive-plate/TPU-dielectric sensor design with 30% dielectric infill (PLA-TPU-30), and a TPU-conductive-plate/TPU-dielectric sensor design with 30% dielectric infill (Eel-TPU-30). The PLA and Eel filaments are made of conductive material and are used to print the sensor electrodes, while nonconductive TPU filament is used as the dielectric infill. Five sensors are printed and evaluated for each of the three sensor types. The cost of each sensor is estimated to be approximately 0.36 USD/sensor for PLA-TPU sensors and 0.48 USD/sensor for Eel-TPU sensors, demonstrating the low cost of the sensors. The sensing system consists of the 3D printed sensors paired with a commercial capacitance-to-voltage converter board. The sensors are evaluated through a series of cyclic compression tests of varying frequencies and pressures. The pressure cycles have a minimum preload pressure of 14.33 N/cm^2^ and the maximum pressure value is varied between 23.88, 33.43, 52.54, and 71.65 N/cm^2^, thus encompassing roughly twice the maximum normal pressure experienced during gait. The frequencies tested are 1, 3, 5, and 7 Hz, which are in the typical range for gait analysis. Results show that average sensitivity varies slightly over the tested pressure and frequency range. The range of average sensitivities for the PLA-TPU-10, PLA-TPU-30, and Eel-TPU-30 sensors over the tested pressures and frequencies are $11.61–20.11\cdot 10^{-4},\;3.30–4.42\cdot 10^{-4},\;\mathrm{and}\;4.85–6.40\cdot 10^{-4}\;V/(N/cm^{2})$, respectively. The PLA-TPU-10 sensors demonstrated the highest average sensitivities, which are 2–4 times greater than the average sensitivities of the PLA-TPU-30, and Eel-TPU-30 sensors, but the PLA-TPU-10 sensitivities also have the greatest variability. Additionally, all sensor types exhibit significant hysteresis and nonlinearity. The range of average hysteresis values for PLA-TPU-10, PLA-TPU-30, and Eel-TPU-30 sensors are 11.9–31.8, 33.4–60.9, and 24.3–51.7%, respectively, and the range of average linearity error for PLA-TPU-10, PLA-TPU-30, and Eel-TPU-30 sensors are 9.0–22.3, 24.6–43.5, and 18.0–36.5%, respectively. Both hysteresis and linearity error for all sensor types tend to decrease with an increase in pressure and have little dependence on frequency. The PLA-TPU-10 sensors showed significantly lower average hysteresis and linearity error when compared to PLA-TPU-30 and Eel-TPU-30 sensor types. The significant hysteresis and linearity error are due to the viscoelastic properties of TPU, and some additional nonlinear effects may be due to buckling of the infill walls of the dielectric. It should be noted that the signal-to-noise ratio (SNR) is low for the smallest pressure levels tested, thus there is more uncertainty in the quantification of hysteresis and linearity error at these pressures, which in turn increases the maximum hysteresis and linearity errors reported above. While the hysteresis and linearity error are high across the full range of tested frequencies and pressures, these errors reduce over narrower input ranges, and specific performance limits on hysteresis or linearity error would be application-driven. Overall, the PLA-TPU-10 sensor showed the most promising results for application in customizable pressure-sensing insoles due to the highest average sensitivity value together with the lowest average hysteresis and linearity errors.

This work has several limitations, and there are opportunities for future sensor development work. Efforts may be made to increase the sensitivity of the sensors; however, nonlinearities and hysteresis will still exist due to the inherent viscoelastic nature of the flexible TPU dielectric. The PLA-TPU-10 sensors, despite performing the best, are not sensitive enough to measure down to 1.5 N/cm^2^, which is typical for commercial pressure-sensing insoles. Therefore, the current sensors are not yet sensitive enough to be used in footbed pressure-sensing applications. Due to the significant hysteresis and nonlinearity, each sensor would either require a lookup table or a nonlinear and hysteretic model to be useful for footbed pressure-sensing applications. The variation in sensitivity also demonstrates that even under the controlled 3D printing environment presented in this work, the sensor quality and properties still varied significantly. Therefore, in the current work, each sensor would need to be characterized individually, which is not practical for footbed pressure-sensing applications where sensors are printed within an insole. Future work will focus on improving methods for printing flexible filament consistently, such as modifying the direct-drive extruder component of the LulzBot TAZ 6 printer so that the filament would be extruded at a more consistent rate. Improving print consistency would reduce variability in the sensors and the need for full characterization of each sensor inside an insole. Additionally, some of the variability in sensor behavior comes from poor print quality at low infill densities. Future experiments will investigate if a more flexible TPU filament could be used to print more sensitive sensors with a higher infill percentage having less variability. Other work will include designing and testing a conductive PLA filament with a higher conductivity, which could increase the output voltage range of the flexible sensors in this work. Finally, there is a general lack of documentation for the SingleTact capacitance-to-voltage converter board concerning (a) its method of measuring capacitance, (b) its self-calibration process, including how it sets its sensitivity, (c) any potential contributions to nonlinearity and hysteresis, and (d) any susceptibility to noise. Any of these unknowns could negatively influence the sensor data collected and analyzed in this study. Future work will focus on the development of a custom-designed and well-characterized circuit to convert between capacitance and voltage.

## Figures and Tables

**Table 1:** Chosen print parameters for each filament used.

Setting	NinjaTek NinjaFlex TPU	NinjaTek Eel TPU	Protopasta Conductive PLA
Layer Height	0.125 mm	0.125 mm	0.125 mm
Initial Layer Height	0.3 mm	0.3 mm	0.3 mm
Infill Pattern	Grid	Lines	Lines
Infill Density	10% or 30%	100%	100%
Infill Overlap Percent	15%	15%	75%
Skin Overlap Percent	15%	15%	75%
Printing Temperature	230 °C	230 °C	215 °C
Build Plate Temperature	41 °C	41 °C	41 °C
Flow	150%	150%	130%
Initial Layer Flow	150%	150%	130%
Enable Retraction	Unchecked	Unchecked	Unchecked
Standby Temperature	230 °C	230 °C	230 °C
Print Speed	9 mm/s	9 mm/s	9 mm/s
Infill Speed	9 mm/s	9 mm/s	35 mm/s
Wall Speed	9 mm/s	9 mm/s	9 mm/s
Outer Wall Speed	9 mm/s	9 mm/s	30 mm/s
Inner Wall Speed	9 mm/s	9 mm/s	9 mm/s
Enable Print Cooling	Unchecked	Unchecked	Unchecked
Enable Prime Tower	Unchecked	Unchecked	Unchecked
Enable Ooze Shield	Checked	Checked	Checked
Ooze Shield Angle	0°	0°	0°
Ooze Shield Distance	6 mm	6 mm	6 mm

**Table 2:** Amplified Omega load cell sensitivity and intercept across the range of frequencies investigated.

Frequency (Hz)	Sensitivity (mV/N)	Intercept (mV)
1	0.331	−2.48
3	0.332	−2.95
5	0.340	−1.35
7	0.347	−2.34

**Table 3:** Average sensing system output voltage range and SNR.

Frequency (Hz)	Pressure (N/cm^2^)	Average Output Voltage Range (V)			Average SNR (dB)		
		PLA-TPU-10	PLA-TPU-30	Eel-TPU-30	PLA-TPU-10	PLA-TPU-30	Eel-TPU-30
1	23.9	0.090	0.055	0.059	9.060	3.844	5.076
1	33.4	0.106	0.055	0.061	13.761	4.982	7.674
1	52.5	0.179	0.057	0.092	18.603	8.004	10.400
1	71.7	0.275	0.071	0.097	22.281	10.749	13.501
3	23.9	0.090	0.049	0.073	9.447	3.332	5.081
3	33.4	0.103	0.051	0.061	12.709	5.641	7.418
3	52.5	0.157	0.076	0.078	15.688	7.322	9.768
3	71.7	0.248	0.070	0.107	21.145	9.239	12.697
5	23.9	0.084	0.051	0.057	7.952	3.262	3.335
5	33.4	0.108	0.062	0.068	12.588	4.427	4.827
5	52.5	0.148	0.059	0.084	16.575	6.817	9.999
5	71.7	0.210	0.066	0.088	19.970	8.491	11.833
7	23.9	0.078	0.051	0.061	8.149	2.519	5.346
7	33.4	0.098	0.057	0.065	11.312	4.612	6.331
7	52.5	0.152	0.053	0.080	13.716	5.942	9.114
7	71.7	0.211	0.072	0.084	19.205	7.961	10.882

**Table 4:** Average sensitivity, hysteresis, and linearity error.

Frequency (Hz)	Pressure (N/cm^2^)	Average Sensitivity (V/(N/cm^2^)) × 10^−4^			Average Hysteresis (%)			Average Linearity Error (%)		
		PLA-TPU-10	PLA-TPU-30	Eel-TPU-30	PLA-TPU-10	PLA-TPU-30	Eel-TPU-30	PLA-TPU-10	PLA-TPU-30	Eel-TPU-30
1	23.9	14.49	4.42	6.30	31.16	60.87	50.00	22.12	43.52	36.54
1	33.4	15.10	4.35	6.40	24.27	51.86	44.95	17.56	36.79	32.67
1	52.5	18.12	4.32	6.29	14.53	40.29	30.89	10.75	29.84	22.19
1	71.7	20.11	4.27	6.15	11.87	33.41	24.26	9.02	24.64	18.01
3	23.9	13.14	3.94	5.60	31.75	59.56	51.65	22.25	41.53	35.91
3	33.4	13.35	3.87	5.90	25.82	52.44	39.68	18.11	36.37	28.31
3	52.5	15.48	3.82	5.56	19.92	40.05	30.54	14.57	27.79	21.96
3	71.7	16.92	3.75	5.65	17.89	36.84	25.78	13.72	25.59	18.78
5	23.9	12.91	3.59	5.45	29.29	60.08	49.72	21.42	42.07	35.44
5	33.4	12.99	3.47	5.28	23.49	47.52	40.12	19.48	33.99	27.05
5	52.5	14.29	3.38	5.31	20.09	42.07	29.01	17.44	29.67	20.74
5	71.7	14.56	3.60	5.36	18.21	34.02	24.32	15.68	24.74	18.30
7	23.9	12.33	3.69	4.92	30.32	60.29	47.75	22.30	42.17	33.79
7	33.4	11.61	3.33	4.92	25.18	55.55	40.54	20.10	37.96	28.45
7	52.5	13.03	3.31	5.14	23.07	40.69	27.88	17.94	27.94	19.30
7	71.7	14.19	3.30	4.85	22.79	35.41	24.94	17.46	25.13	17.98

**Table 5:** Resolvable pressure and frequency range, along with corresponding sensitivity, hysteresis, and linearity error presented as a range of average values with a 90% confidence interval of deviations about the mean (90% CI), all as a function of minimum SNR.

Minimum SNR (dB)	Resolvable Pressure and Frequency Range (N/cm^2^)/(Hz)			Sensitivity (V/(N/cm^2^)) × 10^−4^			Hysteresis (%)			Linearity Error (%)		
	PLA-TPU-10	PLA-TPU-30	Eel-TPU-30	PLA-TPU-10	PLA-TPU-30	Eel-TPU-30	PLA-TPU-10	PLA-TPU-30	Eel-TPU-30	PLA-TPU-10	PLA-TPU-30	Eel-TPU-30
0	(23.88–71.65)/(1–7)	(23.88–71.65)/(1–7)	(23.88–71.65)/(1–7)	11.61–20.11 (±20%)	3.30–4.42 (±17%)	4.85–6.40 (±14%)	11.9– 31.8(±17%)	33.4–60.9 (±12%)	24.3–51.7 (±13%)	9.0–22.3 (±20%)	24.6–43.5 (±11%)	18.0–36.5 (±11%)
6	(23.88–71.65)/(1–7)	(71.65)/(1–7)	(52.54–71.65)/(1–7)	11.61–20.11 (±20%)	3.30–4.27 (±12%)	4.85–6.29 (±15%)	11.9–31.8 (±17%)	33.4–36.8 (±9%)	24.3–30.9 (±14%)	9.0–22.3 (±20%)	24.6–25.6 (±8%)	18.0–22.2 (±12%)
8	(33.43–71.65)/(1–7)	(71.65)/(1–5)	(52.54–71.65)/(1–7)	11.61–20.11 (±19%)	3.60–4.27 (±12%)	4.85–6.29 (±15%)	11.9–25.8 (±17%)	33.4–36.8 (±10%)	24.3–30.9 (±14%)	9.0–20.1 (±22%)	24.6–25.6 (±8%)	18.0–22.2 (±12%)
10	(33.43–71.65)/(1–7)	(71.65)/(1)	(71.65)/(1–7)	11.61–20.11 (±19%)	4.27–4.27 (±14%)	4.85–6.15 (±15%)	11.9–25.8 (±17%)	33.4–33.4 (±10%)	24.3–25.8 (±13%)	9.0–20.1 (±22%)	24.6–24.6 (±9%)	18.0–18.8 (±10%)

**Table 6:** Comparison of sensors to other 3D printed capacitive pressure/force sensors related to orthopedics.

	Sensor Performance			Evaluation Range		Materials and Fabrication Methods			
Source	Sensitivity	Hysteresis Error	Linearity Error	Tested Frequencies	Tested Pressures	Electrode	Dielectric	Continuously 3D printed	Fully 3D Printed
PLA-TPU-10 (this work)	$11.61–20.11\cdot 10^{-4}$ V/(N/cm^2)	11.9–31.8%	9.0–22.3%	1–7 Hz	23.9–71.7 N/cm^2^	PLA; FDM Printed	TPU; FDM Printed	Yes	Yes
PLA-TPU-30 (this work)	$3.30–4.42\cdot 10^{-4}$ V/(N/cm^2^)	33.4–60.9%	24.6–43.5%	1–7 Hz	23.9–71.7 N/cm^2^	PLA; FDM Printed	TPU; FDM Printed	Yes	Yes
Eel-TPU-30 (this work)	$4.85–6.40\cdot 10^{-4}$ V/(N/cm^2^)	24.3–51.7%	18.0–36.5%	1–7 Hz	23.9–71.7 N/cm^2^	TPU; FDM Printed	TPU; FDM Printed	Yes	Yes
[32]	1190 ${\mathrm{kPa}}^{-1}$ $\left(11,900\;{\left(N/cm^{2}\right)}^{-1}\right)$	9.8%	-	0.5–1.167 Hz	41.5–872.4 kPa $\;(4.15–87.24\;N/cm^{2})$	PLA; FDM Printed	TPU; FDM Printed	No	Yes
[26]	$0.702–7.57\;{\mathrm{kPa}}^{-1}$ $\;(7.02–75.7\;{\left(N/cm^{2}\right)}^{-1})$	-	-	-	0–35 kPa $\left(0–3.5\;N/cm^{2}\right)$	TPU; FDM Printed	EcoFlex Rubber; poured	No	No
[23]	$0.0112–0.101\;{\mathrm{kPa}}^{-1}$ $\left(0.112–1.01\;{\left(N/cm^{2}\right)}^{-1}\right)$	-	-	0.5 Hz	0.3–44 kPa $\left(0.03–4.4\;N/cm^{2}\right)$	AgNW and filter paper; poured and heated	UV resin/TPU; DLP/FDM Printed	No	No
[24]	$0.08–0.5\;{\mathrm{kPa}}^{-1}$ $\left(0.8–5\;{\left(N/cm^{2}\right)}^{-1}\right)$	-	-	-	0.001–0.5 kPa $\left(0.01–5\;N/cm^{2}\right)$	Silver; Ink Jet Printed	9495MP 3M tape; placed by hand	No	No
[33]	854–1065 ${\mathrm{kPa}}^{-1}$ $\left(8540–10,650\;{\left(N/cm^{2}\right)}^{-1}\right)$	9.57%	-	-	0–300 kPa $\left(0–30\;N/cm^{2}\right)$	TPU; FDM Printed	EcoFlex Rubber; poured	No	No

AgNW = silver nanowires.

## Data Availability

Not applicable.
